# The FDA guidance for industry on PROs: the point of view of a pharmaceutical company

**DOI:** 10.1186/1477-7525-4-85

**Published:** 2006-10-31

**Authors:** Fabio Arpinelli, Francesco Bamfi

**Affiliations:** 1 Health Technology Assessment, Medical Department, GSK S.p.A. Verona, Italy

## Abstract

The importance of the patients point of view on their health status is widely recognised. Patient-reported outcomes is a broad term encompassing a large variety of different health data reported by patients, as symptoms, functional status, Quality of Life and Health-Related Quality of Life. Measurements of Health-Related Quality of Life have been developed during many years of researches, and a lot of validated questionnaires exist. However, few attempts have been made to standardise the evaluation of instruments characteristics, no recommendations are made about interpretation on Health-Related Quality of Life results, especially regarding the clinical significance of a change leading a therapeutic approach. Moreover, the true value of Health-Related Quality of Life evaluations in clinical trials has not yet been completely defined. An important step towards a more structured and frequent use of Patient-Reported Outcomes in drug development is represented by the FDA Guidance, issued on February 2006.

In our paper we aim to report some considerations on this Guidance. Our comments focus especially on the characteristics of instruments to use, the Minimal Important Difference, and the methods to calculate it. Furthermore, we present the advantages and opportunities of using the Patient-Reported Outcomes in drug development, as seen by a pharmaceutical company. The Patient-Reported Outcomes can provide additional data to make a drug more competitive than others of the same pharmacological class, and a well demonstrated positive impact on the patient' health status and daily life might allow a higher price and/or the inclusion in a reimbursement list. Applying extensively the FDA Guidance in the next trials could lead to a wider culture of subjective measurement, and to a greater consideration for the patient's opinions on his/her care. Moreover, prescribing doctors and payers could benefit from subjective information to better define the value of drugs.

## Introduction

The importance of the patients point of view on their health status and healthcare is widely recognized [1]. Patient-reported outcomes (PROs) provide the patient's perspective on health outcome endpoint data [2-4]. PROs can play an important role in the development of new drugs, especially those aimed to treat medical conditions in which only subjective data allow to evaluate the treatment effect [1].

PROs is a broad term encompassing a large variety of different health data reported by patients. PROs as symptoms, functional status, treatment adherence, satisfaction with care represent useful data to corroborate the clinical data (efficacy and safety), helping clinicians to better define the drug profile.

Furthermore, inside the PROs we meet a couple of important concepts, sometimes considered as synonymous. These concepts are the Quality of Life (QoL) and the Health-Related Quality of Life (HRQoL). The QoL is a complex, abstract, multidimensional concept defining an individual satisfaction with life in domains he/she considers important. The HRQoL reflects an attempt to restrict the complex concept of QoL to those aspects of life specifically related to the individual health, and potentially modified by healthcare [5]. HRQoL data are not always foreseeable and necessarily correlated with the severity of the disease as perceived by healthcare professionals. Moreover, the symptoms/HRQoL correlation could be weak (for example, no abdominal pain during a medical examination but a poor patient's HRQoL, because of the impairment of the patient personal life and leisure, his/her need to take drugs, dietary restrictions etc.).

HRQoL measurements has been developed during many years of research (proven by thousand of published papers), and a lot of validated questionnaires exist, both generic and disease specific. However, the following points need to be considered: 1) although the operational application of concepts and their validation process have been well codified, few attempts have been made to standardise the evaluation of instruments characteristics; 2) usually, the criteria regard intrinsic characteristics of the questionnaires (reliability, validity etc.), while no recommendations are made about interpretation on HRQoL results, especially regarding the clinical significance of a change in HRQoL leading a therapeutic approach; 3) despite some scientific society have created working groups to debate the role of HRQoL in clinical research, the true value of HRQoL evaluations in clinical trials has not yet been completely defined [5,8,9].

The contribution given by the PROs measurement could be important in the process of drug approval by regulatory authorities. Furthermore, on the regulatory side some factors limit the use of PROs, and HRQoL in particular. The main limiting factors are: 1) the abuse of the term HRQoL in clinical trials. This term is used also when other PROs are measured (symptoms, drug side effects etc.). 2) The poor quality of the majority of clinical trials having the HRQoL as primary endpoints. 3) The role and the significance of HRQoL as efficacy, tolerance, utility endpoint [10,11]

These points and the reasonable scepticism of regulatory authorities to officially acknowledge some subjective criterion whose clinical meaning remains difficult, have limited the use of PROs in the drugs approval process. At the moment, it could be quite difficult to make acceptable HRQoL to regulatory authorities as a primary endpoint, since some regulators consider it as a less rigorous secondary endpoint.

An important step towards a more structured and frequent use of PROs in drug development has been done by FDA. On February 2006 the FDA issued the Guidance, that describes how it evaluates PROs used as effectiveness endpoints in clinical trials.

## Specific comments to the Guidance

The Guidance is potentially very useful for all concerned in planning, designing and carrying out clinical trials for regulatory purposes. It provides information on how to choose a PRO instrument. Although the Guidance is clear enough and take into consideration a lot of important topics on PRO instruments (their development, assessment of measurement properties, modification of existing instruments), study design and data analysis, it could be improved to make it more applicable to NDA trials and facilitate univocal interpretation of results by experts and regulators.

The reading of the FDA Guidance firstly led to some general considerations and comments on the PROs. We aim to briefly report these considerations.

PROs is an "umbrella term". It contains physical functioning, psychological well-being, global health perception, treatment satisfaction and other subjective outcomes. Therefore, PRO is not interchangeable with QoL or HRQoL.

QoL has never been approved in a labelling claim because of its vagueness. On the contrary, HRQoL could be a possible endpoint. This should be very clear when measuring PROs.

The inclusion of PROs assessment in clinical trials should have a good scientific rationale. The risk of an indiscriminate measuring of PROs is producing useless and confounding data.

The conceptual framework of a single-item symptom measure is not so complex as a multiple frameworks to define HRQoL. Multiple domains questionnaires usually are required in early phases of drug development, when researchers investigate the activity of a new compound more than its efficacy. In this phase the need to focus the attention on the domains more affected by the disease (or by the disease management) makes useful a multi-domains questionnaire. In later phases, when needs and expectations of pts are well known, a single or few domains questionnaire helps to a better interpretation of changes.

HRQoL measurements is more useful in chronic diseases (for example rheumatoid arthritis) than in life-threatening disease (cancer). In life-threatening diseases the only acceptable main aim of the therapy is a longer survival. A better HRQoL and a worsened survival make that drug probably not approved by regulatory authorities.

PROs should not replace safety reporting, as safety is an important concern of regulatory authorities.

Researchers aiming to measure HRQoL must use existing, validated instruments. The development of new questionnaires should be discouraged, but the standardisation of questionnaires should be encouraged. In particular, the development of a questionnaire for a certain study should be definitely avoided. The sponsor of the study has to provide evidence of validity of the selected instrument (for example, a list of published papers on the development, validation and use in clinical trials of the questionnaire).

When an existing questionnaire is used in a new population (elderly rather than adults) or in a different context (on outpatients basis rather than inpatients), a re-validation is required.

The instrument used to measure a PRO should have a documented evidence of responsiveness/sensitivity to changes in health status. In fact, small differences in PRO scores, although statistically significant, are often questioned with regard to their clinical importance. It is not always clear what is meant by clinically importance, i.e., discernible to the patient, significant enough for a clinician to change an intervention, or significant from a population perspective. Hence if a PRO cannot detect a meaningful change in health status, its use may be risky, because clinically meaningful effects may be undetected [2,6].

Demonstrating responsiveness is necessary to determine the Minimal Important Difference (MID), where MID represents the smallest change perceived by the patient as an advantage, or that could lead to a change of treatment [6].

The MID can be calculated using a number of anchor-based or distribution-based methods. Distribution-based approaches are the effect size (ES), the standardised response mean and the standard error of measurement (SEM). Anchor-based methods assess which changes on the measurement instrument correspond with a minimal important change defined on a anchor. Distribution-based methods do not provide a good indication of the importance of the observed change. Anchor-based approaches do not take measurement precision into account. Sometimes results obtained using these different approaches are similar [12].

At the moment, there is not a clear agreement on the recommended, best practice approach for determining the MID [7]. The application of multiple methods, even if imperfect, to the same datasets could tend to give similar results and this should clarify the relationship between these methods and give a better estimate of the MID. Furthermore, some Authors report that for assessing the MID anchor-based methods are preferred, as they include a definition of what is minimally important [12].

These concepts should be more stressed in the Guidance. The MID may vary by context, and different MID could be valid for different studies where PROs instruments are used. MID varies according different factors, such as the underlying disease, the characteristics of the population, the healthcare scenario, and so on. For these reasons, we cannot have a unique MID for a PRO instrument, good for different diseases and patients [7]. It is necessary that responsiveness and MID be well documented in order to use PROs in labelling claims.

The patient satisfaction is a PRO, but it could be greatly influenced by factors such as, for example, the personal relationship between the patient and the nurse/doctor. This relationship can satisfy/dissatisfy the patient, and represents an aspect related to the (variable) healthcare structure/organization. For this reason we believe that the patient satisfaction should be considered as a less important indicator than HRQoL.

The users of the Guidance should appreciate more details for sample size determination and handling missing data, especially for the questionnaire development. Another topic to be detailed is concerned with the proxy measures.

Furthermore, we are aware that the heterogeneity of clinical settings, diseases and drugs makes very difficult to anybody (including FDA) to prepare a technical documentation applicable to any context.

## The point of view of a pharmaceutical company

Certainly the drug developers are interested in a better definition of the value of their drugs using PROs data. The pharmaceutical industry has been the principal driving force behind the expansion in the number and type of HRQoL instruments available to clinician and researchers [13].

Industry sees some advantages in PRO measurements. Infact, PROs can provide additional data for inclusion of a drug in a formulary, making that drug more competitive than others of the same pharmacological class. Furthermore, an effective and well tolerated drug with a demonstrated positive impact on the patient' health status and daily life might allow to negotiate a higher price (where the price of drugs is negotiated between pharmaceutical companies and regulatory authorities) and/or the inclusion in a reimbursement list.

Subjective data collection has to be regulated by clear rules, agreed by all parts involved in the development and approval of drugs. The Guidance is a positive and modern attempt to provide a document helping the use of subjective data to support labelling claims. It stimulates pharmaceutical companies to use a shared and accepted methodology to provide data, although an alternative approach is considered possible. This means longer time to prepare and carry out a clinical trial, and more expenses. On the other side, the adherence to the Guidance should reduce the risk of rejection of PROs data by FDA.

Furthermore, the Guidance offers a good opportunity to the pharmaceutical industry to discuss about methodology with regulatory authorities, and to become a trustworthy partner of regulatory agencies.

The development of a new questionnaire (if needed) or their revision/updating is a complex, time consuming and expensive activity. Usually a pharmaceutical company can provide financial support and technical knowledge to develop subjective questionnaires by itself or in partnership with a scientific society and academic experts. Adequate resources can allow tool developers to reach an exhaustive set of data to demonstrate the validity and reliability of questionnaires. A further important step should be the publication of papers, allowing the developers to insert the new tools in a compendium, where all concerned researchers and regulators can find the instruments and replicate experiences to confirm the validity of the instruments.

Regulatory authorities might recognise that the development of a new instrument allows clinicians to have a useful instrument to administer to their patients. The companies could waive the copyright in favour of all researchers and clinicians, obtaining, on the other hand, both an increase of robustness of the tool and, maybe, a reward by regulators.

A wider use of PRO measurements allows clinicians/payers to become familiar with PROs, integrating these data in their evaluation criteria to prescribe or reimburse a drug.

## Conclusion

In conclusion, it is well known that the correlation between the patient and the physician evaluation of a certain symptom could be poor and not univocal. HRQoL and other PROs provide important patient perspective on disease and the treatment they receive. A subjective evaluation provides clinically important information not captured by objective measures. This is particularly important in chronic diseases, as rheumatoid arthritis or asthma, where HRQoL data capture the overall benefit given by the treatment.

Despite a very large number of published papers on HRQoL, there is a certain scepticism on the value of HRQoL and other PROs. It is likely that clinicians do not use PROs because they are not routinely trained in the use and interpretation of PRO instruments [1]. Usually, the interpretation of the clinical significance of a change in HRQoL is considered difficult; particularly difficult is the translation of results into an overall clinical evaluation leading to a change of the current therapy [14,15].

In order to overcome this scepticism, it is necessary to highlight the scientific and statistical basis of these measurements, and the usefulness of collecting these data. Moreover, it shall be demonstrated the improvement of patients management by clinicians thanks to the use of PROs data.

The use of these data to support a labelling claim requires the use of a rigorous methodology, based on valid and reliable instruments, used when appropriate.

A parallel European Guidance has not yet been conceived by EMEA, and this is not surprising considering the differences between the American and European healthcare and regulatory structures. This is reflected in a different marketing approval process, which is first centrally granted (EMEA), and subsequently discussed at the national level (reimbursement, price). Furthermore, EMEA prepared a Reflection Paper (July 2005), a short and generic document that discusses the place that HRQoL may have in drug evaluation process, and gives some broad recommendations.

The FDA guidance represents the first step in a hard, complex track to reach the best evidence in questionnaire development and the use of PRO to support labelling claims.

Applying extensively the Guidance in the next trials could lead to a wider culture of subjective measurement, and to take into a greater consideration the patient's point of view on his/her care. Moreover, a more detailed evaluation of drugs is helpful for prescribing doctors and payers, to allow them to better define the value of drugs.

## Competing interests

The author(s) declare that they have no competing interests.

## Authors' contributions

FA discussed the FDA guidance, exposed the point of view of a pharmaceutical company and drafted the manuscript. FB reviewed the manuscript in order to improve the parts that deal with statistics.
